# Repetitive transcranial magnetic stimulation for freezing of gait in Parkinson's disease: a systematic review and meta-analysis

**DOI:** 10.1016/j.prdoa.2026.100465

**Published:** 2026-06-15

**Authors:** Lara Hamzeh Hamzeh, Yahya Kayed AbuJwaid, Sara M.F. Fahmy, Dania AbuHawas, Habiba Tariq Saeed, Daher Heib, Mawatheeq Al-Yafrosi, Ahmed Noureldeen Abbas, Karima El Refaei, Christian Cortes Armijo, Majd A. AbuAlrob

**Affiliations:** a Faculty of Medicine, Caucasus International University, Tbilisi, Georgia; b Faculty of Medicine, Al-Quds University, Jerusalem, Palestine; c Faculty of Medicine, Delta University for Science and Technology, Al Mansurah, Dakahlia, Egypt; d University of Jordan, Amman, Jordan; e Faculty of Medicine, Tanta University, Egypt; f Faculty of Medicine and Health Sciences, University of Aden, Yemen; g Faculty of Medicine, Minia University, Egypt; h School of Medicine, Newgiza University (NGU), Giza, Egypt; i Facultad de Medicina, Universidad Autónoma de Nuevo León, Mexico; j Neurology Department, Hamad Medical Corporation, Doha, Qatar

**Keywords:** Parkinson's disease, Freezing of gait, Repetitive transcranial magnetic stimulation, Neuromodulation, Systematic review, meta-analysis

## Abstract

**Background:**

Freezing of gait is among the most disabling motor complications of Parkinson's disease, markedly increasing fall risk and reducing quality of life. Repetitive transcranial magnetic stimulation has emerged as a non-invasive neuromodulation approach; however, evidence regarding its efficacy for freezing of gait remains heterogeneous and inconclusive.

**Objectives:**

To evaluate the efficacy and safety of repetitive transcranial magnetic stimulation for freezing of gait and motor outcomes in Parkinson's disease.

**Methods:**

A systematic review and meta-analysis was conducted following Preferred Reporting Items for Systematic Reviews and Meta-Analyses 2020 guidelines (PROSPERO: CRD420261285716). PubMed, Scopus, Cochrane Library, and Web of Science were searched through January 2026 for randomized controlled trials. The primary outcome was freezing of gait severity measured by the Freezing of Gait Questionnaire. Pooled standardized mean differences were calculated using random-effects modeling.

**Results:**

Twelve randomized controlled trials comprising 293 participants were included. Repetitive transcranial magnetic stimulation significantly improved freezing of gait severity as measured by the Freezing of Gait Questionnaire (standardized mean difference = −0.97, 95% confidence interval − 1.39 to −0.55; *p* < 0.001). Significant improvements were also observed in motor function, Timed Up and Go performance, gait speed, step/stride length, turn time, and turn steps, whereas cadence did not differ significantly. No serious adverse events were reported.

**Conclusions:**

Repetitive transcranial magnetic stimulation significantly improves freezing of gait and motor performance in Parkinson's disease, representing a safe and promising adjunctive therapy for gait disturbances refractory to conventional treatment. Future large-scale trials with standardized protocols are needed to confirm long-term efficacy.

## Highlights

• rTMS significantly reduces FOG severity in Parkinson's disease (SMD = −0.97).
• SMA and M1 are the most effective cortical stimulation targets for FOG.
• Both low-frequency (1 Hz) and high-frequency (10 Hz) protocols are effective.
• rTMS improves motor function, gait speed, TUG, and turning parameters.
• No serious adverse events were reported across 12 randomized trials.

ORCID: https://orcid.org/0000-0002-9707-9266

## 1 Introduction

Parkinson's disease (PD) is a chronic, progressive neurodegenerative disorder characterized by the loss of dopaminergic neurons in the substantia nigra, leading to motor dysfunction [1]. It is the second most common age-related neurodegenerative disorder and one of the most prevalent movement disorders worldwide. According to the 2021 Global Burden of Disease study, approximately 11.77 million people are living with Parkinson's disease **(PD)** globally, and this number is projected to double by 2050. Parkinson's disease **(PD)** is 1.5 times more common in males than females [2], [3], [4]. The etiology of Parkinson's disease **(PD)** is multifactorial, arising from complex interactions between genetic susceptibility, environmental exposures, and aging. These pathophysiological changes disrupt neural circuits involved in movement initiation and coordination, resulting in a wide range of motor and non-motor symptoms [5]. The cardinal motor features include tremor, rigidity, bradykinesia, and postural instability, while non-motor manifestations include depression, anosmia, constipation, and sleep disturbances [6].

Gait disturbances become more prominent as the disease progresses and represent a major source of disability. Among these, freezing of gait (FOG) is a particularly disabling phenomenon characterized by a transient inability to initiate or maintain walking [7]. Freezing of gait **(FOG)** commonly occurs in advanced stages of Parkinson's disease **(PD)** and is strongly associated with increased risk of falls, injuries, functional dependence, and reduced quality of life [7], [8]. Management of Parkinson's disease **(PD)** is primarily symptomatic and aims to improve functional status and quality of life. Therapeutic approaches include pharmacological treatment, physiotherapy, occupational therapy, speech therapy, and surgical interventions [9]. Levodopa, a dopamine precursor, remains the cornerstone of treatment and is highly effective in alleviating cardinal motor symptoms. However, as Parkinson's disease **(PD)** progresses, many patients develop motor complications, including motor fluctuations, wearing-off phenomena, and dyskinesia; the extent to which these complications reflect levodopa exposure versus disease duration and progression remains debated [10]. Notably, axial symptoms such as postural instability and gait disturbances, including freezing of gait **(FOG)**, often respond poorly to dopaminergic therapy, highlighting a critical unmet clinical need.

Repetitive transcranial magnetic stimulation (rTMS) has emerged as a promising non-invasive neuromodulation technique that can modulate cortical excitability through targeted magnetic stimulation of specific brain regions [11], [12]. By influencing motor network activity, rTMS has the potential to improve gait dysfunction in patients with Parkinson's disease **(PD)**. Although previous studies have suggested potential benefits of rTMS for gait disturbances in Parkinson's disease **(PD)**, the evidence remains heterogeneous due to variability in stimulation parameters, target regions, and study designs. Moreover, prior systematic reviews have not consistently focused on freezing of gait as a primary outcome. Therefore, this updated systematic review and meta-analysis aims to provide the most comprehensive and current evidence on the efficacy and safety of rTMS specifically for freezing of gait **(FOG)** in patients with Parkinson's disease **(PD)**.

## 2 Methods

### 2.1 Protocol and registration

This systematic review and meta-analysis was conducted in accordance with the Preferred Reporting Items for Systematic Reviews and Meta-Analyses (PRISMA) 2020 guidelines [13]. The study protocol was prospectively registered in PROSPERO (registration number: CRD420261285716; https://www.crd.york.ac.uk/PROSPERO/view/CRD420261285716).

### 2.2 Search strategy

A comprehensive literature search was performed in PubMed (*n* = 674), Scopus (*n* = 2304), Cochrane Library (*n* = 606), and Web of Science (*n* = 1612) from inception through January 2026. Only studies published in English were included due to feasibility constraints. The search strategy combined MeSH terms and free-text keywords including: (“Transcranial Magnetic Stimulation”[MeSH] OR rTMS[Title/Abstract] OR TMS[Title/Abstract]) AND (“Parkinson's disease”[MeSH] OR “PD”[Title/Abstract]) AND (“Freezing of gait”[Title/Abstract] OR “Gait”[Title/Abstract]). Additional studies were identified by manual screening of reference lists from previous systematic reviews. The final search was conducted on January 31, 2026. An updated search was subsequently performed on May 8, 2026 to identify any newly published eligible randomized controlled trials from the early months of 2026. Two independent reviewers performed the literature search and screening process.

### 2.3 Eligibility criteria

Studies were included if they met the following PICOS criteria: Population: adults with Parkinson's disease **(PD)** experiencing freezing of gait **(FOG)**; Intervention: rTMS; Comparison: sham rTMS, usual care, or no intervention; Outcomes: primary outcome was change in freezing of gait **(FOG)** severity measured using the Freezing of Gait Questionnaire (FOG-Q), with secondary outcomes including motor function (UPDRS Part III or MDS-UPDRS Part III), Timed Up and Go (TUG), gait speed, step/stride length, cadence, turn time, turn steps, and adverse events; Study design: randomized controlled trials (RCTs). Studies were excluded if they were: (a) duplicates; (b) non-RCTs; (c) unavailable in full text; (d) Studies were excluded if they did not report extractable FOG-specific outcome data, including FOG-Q, NFOG-Q, or equivalent freezing-of-gait measures; (e) contained unextractable outcome data; or (f) conference abstracts without full peer-reviewed publications.

### 2.4 Study selection and data extraction

All retrieved records were imported into Rayyan software for screening, and duplicates (*n* = 1792) were removed. The remaining 3404 records were screened based on title and abstract, and 31 full-text articles were assessed for eligibility. Two independent reviewers screened titles and abstracts, followed by full-text assessment. Disagreements were resolved through discussion or consultation with a third reviewer. Extracted data included: study and author information; participant characteristics (age, sex, disease duration, Hoehn and Yahr stage); intervention details (stimulation site, frequency, intensity, number of sessions, pulses per session); control conditions; outcome measures; sample size; and follow-up duration. Data extraction was performed independently by two reviewers using a standardized form, and discrepancies were resolved through discussion.

### 2.5 Risk of Bias assessment

The risk of bias of included RCTs was assessed using the Cochrane Risk of Bias 2 (RoB 2) tool [14]. The following domains were evaluated: bias arising from the randomization process, bias due to deviations from intended interventions, bias due to missing outcome data, bias in measurement of the outcome, and bias in selection of the reported result. Each study was classified as having low risk of bias, some concerns, or high risk of bias. Two reviewers independently assessed risk of bias, with disagreements resolved by consensus.

### 2.6 Data synthesis and statistical analysis

Meta-analyses were performed using Comprehensive Meta-Analysis software (CMA, version 4) with the DerSimonian–Laird random-effects model [15]. Continuous outcomes were pooled using standardized mean differences (SMD) with 95% confidence intervals (CI). Statistical heterogeneity was assessed using Cochran's Q test and quantified using the I^2^ statistic, interpreted as low (<25%), moderate (25–75%), or substantial (>75%) [16]. A *p*-value <0.05 was considered statistically significant. Negative SMD values were defined as favoring rTMS. For cross-over trials, data from the first period were used when available; when only combined data were available, appropriate standard error adjustments were applied [17]. For multi-arm trials including more than one eligible rTMS intervention arm and a shared sham/control group, each eligible intervention arm was included as a separate comparison. To avoid double-counting of the shared control group, the control sample size was divided proportionally across comparisons in accordance with Cochrane Handbook recommendations. No single stimulation arm was preferentially selected when multiple eligible rTMS protocols were reported.

### 2.7 Subgroup, sensitivity, and publication Bias analyses

Subgroup analyses were conducted based on stimulation site (supplementary motor area **(SMA)**, primary motor cortex **(M1)**, non-motor cortical targets) and stimulation frequency (1 Hz, 10 Hz, and a 50 Hz category, the latter comprising conventional 50 Hz rTMS and intermittent theta-burst stimulation [iTBS], which delivers 50 Hz triplets repeated at 5 Hz). Sensitivity analyses were performed using a leave-one-out approach, with an additional analysis excluding cross-over trials for the primary outcome. Publication bias was assessed for outcomes with at least ten contributing studies using funnel plot visual inspection and Egger's regression test [18].

Trial sequential analysis (TSA) was performed for the primary outcome (FOG-Q change score) to evaluate the risk of random error and determine whether the cumulative evidence reached the required information size (RIS). TSA was conducted using a two-sided α of 5%, power of 80%, and a random-effects model with heterogeneity adjustment based on the observed I^2^ value.

### 2.8 Certainty of evidence

The certainty of evidence for the primary outcome was assessed using the Grading of Recommendations Assessment, Development and Evaluation (GRADE) framework [19], considering risk of bias, inconsistency, indirectness, imprecision, and publication bias.

## 3 Results

### 3.1 Study selection

**Fig. 1:**
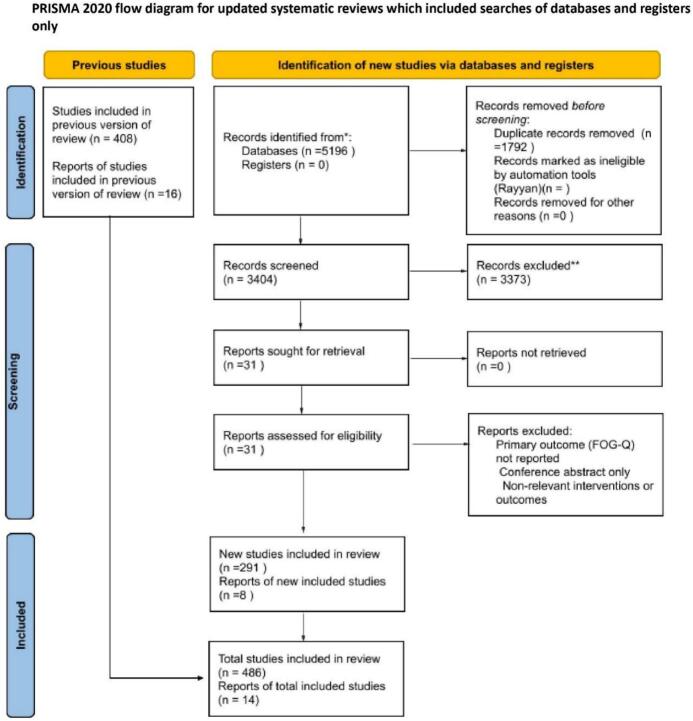
PRISMA 2020 flow diagram of the updated study selection. Abbreviations: PRISMA, preferred reporting items for systematic reviews and meta-analyses; FOG-Q, Freezing of Gait Questionnaire; n, number of records.

The database search identified 5196 records. After removal of 1792 duplicates, 3404 records were screened based on title and abstract, of which 3373 were excluded. Thirty-one full-text articles were assessed for eligibility, of which 12 RCTs met the inclusion criteria and were included in both qualitative and quantitative synthesis. Reasons for full-text exclusion included: conference abstracts without full publication (*n* = 4), registry records or ongoing trials (*n* = 5), duplicates (*n* = 2), non-RCT design (*n* = 1), non-English language (n = 1), no freezing of gait **(FOG)** primary outcome (*n* = 3), and other reasons (n = 3). The study selection process is presented in the PRISMA flow diagram (Fig. 1). An updated search performed on May 8, 2026 did not identify any additional eligible published RCTs. Newly identified records consisted of study protocols, ongoing trials, or secondary evidence syntheses and therefore did not alter the final included study set.

### 3.2 Study characteristics

Twelve RCTs including 293 participants were included [20], [21], [22], [23], [24], [25], [26], [27], [28], [29], [30], [31]. Studies were conducted across China, South Korea, Switzerland, Israel, Canada, Egypt, and the United States. Both parallel-group (*n* = 7) and cross-over (*n* = 5) designs were represented. Mean participant age ranged from the mid-50s to mid-70s, Hoehn and Yahr stages were generally between 2 and 3, and mean disease duration ranged from approximately 4 to 12 years, indicating a predominantly mild-to-moderate to moderately advanced Parkinson's disease **(PD)** population in which freezing of gait (FOG) is clinically relevant and often difficult to treat [7], [8]. Stimulation targets included the primary motor cortex (M1; *n* = 6), supplementary motor area (SMA; *n* = 4), prefrontal cortex (PFC; *n* = 1), and posterior parietal cortex (PPC; n = 1). Stimulation protocols varied substantially across trials in frequency, intensity, number of sessions, pulses per session, and total stimulation dose, with frequencies ranging from 1 Hz to 50 Hz, including intermittent theta-burst stimulation. These protocol-level parameters, together with baseline participant characteristics and medication-state information when reported, are summarized in Table 1.

**Table 1:** Baseline characteristics of the studies included.

Study ID	Country	Year	Design	Technique (Site)	Wave frequency	N	Sex (M/F)	Age (years), mean ± SD	H&Y stage, mean ± SD	Disease duration (years), mean ± SD
Beginner 2012	Switzerland & USA	2012	RCT (Parallel)	rTMS (M1)	50 Hz	13	9/4	55.8 ± 9.1	2.4 ± 0.2	8.06 ± 4.01
				Sham		13	11/2	54.3 ± 12.5	2.5 ± 0.3	9.03 ± 6.08
Brugger 2021	Switzerland	2021	RCT (Crossover)	iTBS and Sham (SMA)	iTBS (50 Hz triplets / 5 Hz)	12	10/2	61.8 ± 13	2.26 ± 0.67	12.67 ± 3.77
Dagan 2017	Israel	2017	RCT (Crossover)	rTMS and Sham (PFC)	10 Hz	7	7/0	74.57 ± 7.09	2.86 ± 0.63	10.29 ± 3.82
Desrochers 2023	canada	2023	RCT (Crossover)	iTBS and Sham (PPC)	iTBS (50 Hz triplets / 5 Hz)	14	8/6	66 ± 13	2.05 ± 0.5	10 ± 5
El-Tamawy 2013	Egypt	2013	RCT (Parallel)	rTMS (M1)	1 Hz	8	11/5 (with Sham group)	67 ± 7.32 (with Sham group)	3.01 ± 0.56 (with Sham group)	NR
				Sham		8				
Kim 2015	korea	2015	RCT (Crossover)	rTMS and Sham (M1)	10 Hz	17	12/5	64.5 ± 8.04	3 ± 0.5	7.08 ± 4.09
Lee 2014	S. Korea	2014	RCT (Crossover)	rTMS and Sham (M1)	10 Hz	20	13/7	71.6 ± 8.06	3.04 ± 0.5	4.07 ± 2.06
Lench 2021	USA	2021	RCT (Parallel)	rTMS (SMA)	1 Hz	12	7/5	66.6 ± 7.05	2.32 ± 0.4	8.07 ± 7.12
				Sham		8	7/1	64.5 ± 8.09	2.29 ± 0.267	8 ± 5.63
Ma 2019	China	2019	RCT (Parallel)	rTMS (SMA)	10 Hz	18	8/10	59.94 ± 9.16	2.42 ± 0.6	8.94 ± 5.48
				Sham		10	5/5	66 ± 8.55	2.4 ± 0.94	7.5 ± 4.72
Mi 2019	China	2019	RCT (Parallel)	rTMS (SMA)	10 Hz	20	9/11	62.65 ± 10.56	2.6 ± 0.85	9.15 ± 5.82
				Sham		10	5/5	65.6 ± 8.68	2.35 ± 0.91	7.4 ± 4.83
Song 2024	China	2024	RCT (Parallel)	rTMS (M1)	10 Hz	22	15/7	67.36 ± 6.99	2.49 ± 0.36	6.18 ± 1.62
				Sham		22	13/9	70.5 ± 6.76	2.52 ± 0.37	6.77 ± 2.02
Zhang 2025	China	2025	RCT (Parallel)	rTMS (M1)	10 Hz	20	13/7	69.05 ± 6.75	2.83 ± 0.4	7.35 ± 2.88
				rTMS (SMA)	10 Hz	20	11/9	67.4 ± 7.22	2.5 ± 0.8	7.05 ± 2.41
				Sham		19	8/11	71.4 ± 6.02	2.83 ± 0.4	7.75 ± 2.57

RCT, randomized controlled trial; M1, primary motor cortex; SMA, supplementary motor area; PFC, prefrontal cortex; PPC, posterior parietal cortex; iTBS, intermittent theta-burst stimulation; H&Y, Hoehn and Yahr.

### 3.3 Risk of Bias assessment

**Fig. 2:**
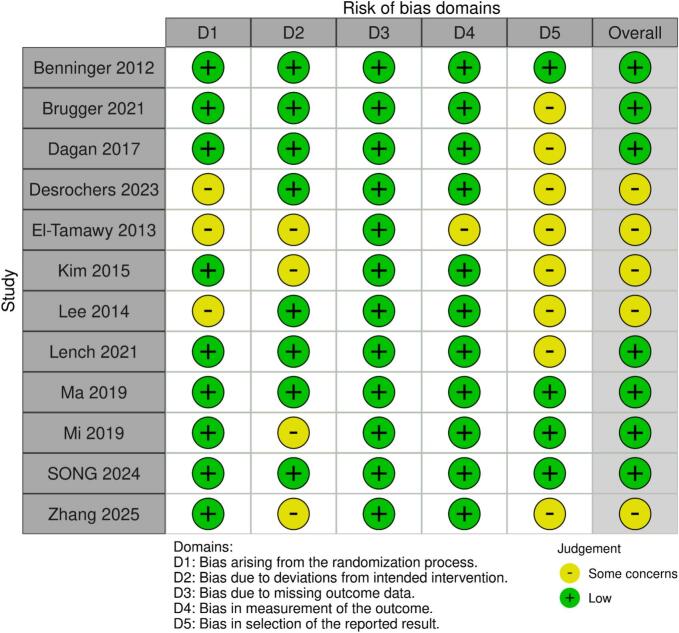
Risk of bias assessment of included randomized controlled trials using the Cochrane Risk of Bias 2 (RoB 2) tool. The traffic-light plot displays the risk of bias judgment for each individual study across five domains: D1, bias arising from the randomization process; D2, bias due to deviations from intended interventions; D3, bias due to missing outcome data; D4, bias in measurement of the outcome; D5, bias in selection of the reported result. Green circles with a plus sign (+) indicate low risk of bias; yellow circles with a minus sign (−) indicate some concerns. No studies were judged to be at high risk of bias in any domain. Overall, seven studies (58.3%) were rated as low risk of bias and five studies (41.7%) as having some concerns. (For interpretation of the references to colour in this figure legend, the reader is referred to the web version of this article.)

Seven studies (58.3%) were judged to have low risk of bias, while five (41.7%) had some concerns. No studies were classified as high risk. Across domains, the proportion judged as low risk was: randomization process (75.0%), deviations from intended interventions (66.7%), missing outcome data (100%), measurement of the outcome (91.7%), and selection of the reported result (33.3%). The risk of bias assessment is summarized in Figs. 2 and Fig. S13.

### 3.4 Primary outcome: FOG-Q change score

**Fig. 3:**
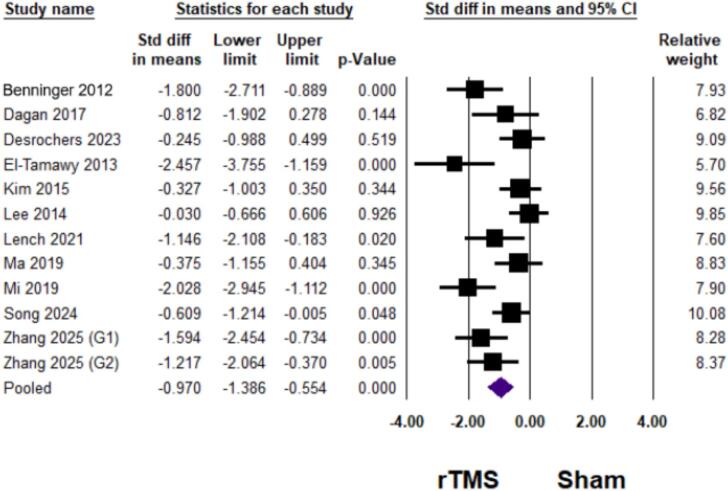
Forest plot of the effect of rTMS on change in FOG-Q scores compared with sham stimulation. Individual study effect sizes are expressed as SMDs with 95% CIs. Negative values favor rTMS. Zhang 2025 (G1) and Zhang 2025 (G2) represent separate intervention arms.

Eleven RCTs with twelve comparisons (Zhang et al. [20] contributed two intervention arms compared with a shared sham control group) were included. The pooled analysis demonstrated a statistically significant improvement in freezing of gait **(FOG)** severity in the rTMS group compared with sham/control (SMD = −0.97, 95% CI −1.39 to −0.55, *p* < 0.001). Moderate-to-substantial heterogeneity was observed (Q = 33.61, p < 0.001; I^2^ = 67.27%). The forest plot is presented in Fig. 3.

### 3.5 Secondary outcomes

#### 3.5.1 Motor function (UPDRS part III)

**Fig. 4:**
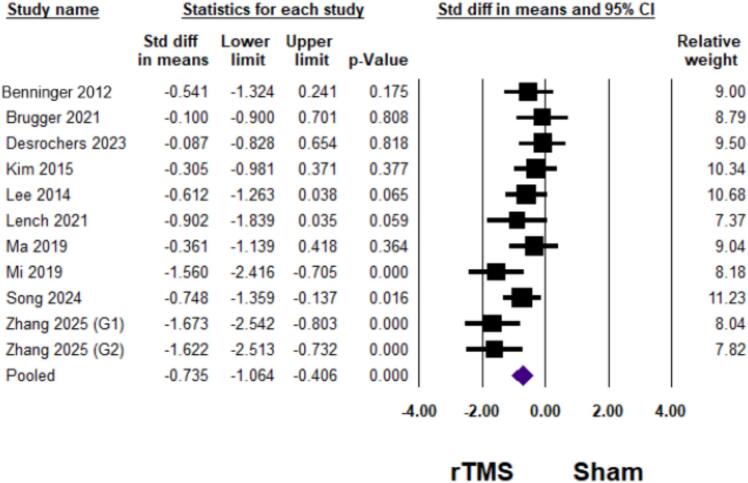
**Forest plot of the effect of repetitive transcranial magnetic stimulation (rTMS) on motor symptoms measured by UPDRS Part III motor scores compared with sham stimulation.** Individual study effect sizes are expressed as standardized mean differences (SMDs) with 95% confidence intervals (CIs). Negative values favor rTMS. Zhang 2025 (G1) and Zhang 2025 (G2) represent separate intervention arms from the same study compared with the shared sham control group.

Eleven RCTs were included. rTMS significantly improved motor scores (SMD = −0.74, 95% CI −1.06 to −0.41, p < 0.001; I^2^ = 50.18%). The forest plot is presented in Fig. 4.

#### 3.5.2 Timed up and go (TUG)

Six RCTs were included. rTMS significantly improved TUG performance (SMD = −0.68, 95% CI −1.25 to −0.11, *p* = 0.020; I^2^ = 72.81%). The forest plot is presented in Fig. S14.

#### 3.5.3 Gait speed

Four RCTs were included. rTMS significantly improved gait speed (SMD = −0.52, 95% CI −0.93 to −0.11, *p* = 0.013; I^2^ = 0%). The forest plot is presented in Fig. S15.

#### 3.5.4 Step/stride length

Three RCTs were included. rTMS significantly improved step/stride length (SMD = −0.54, 95% CI −1.00 to −0.08, *p* = 0.021; I^2^ = 0%). The forest plot is presented in Fig. S16.

#### 3.5.5 Cadence

Three RCTs were included. No significant difference was observed (SMD = −1.79, 95% CI −3.84 to 0.26, *p* = 0.087; I^2^ = 92.23%). Considerable heterogeneity limits interpretability. The forest plot is in Supplementary Fig. S1.

#### 3.5.6 Turn time

Nine RCTs were included. rTMS significantly improved turn time (SMD = −0.86, 95% CI −1.30 to −0.42, *p* < 0.001; I^2^ = 63.66%). The forest plot is presented in Fig. S17.

#### 3.5.7 Turn steps

Six RCTs were included. rTMS significantly reduced turn steps (SMD = −0.42, 95% CI −0.70 to −0.14, *p* = 0.004; I^2^ = 0%). The forest plot is presented in Fig. S18.

### 3.6 Subgroup analysis

By stimulation site, significant effects were observed for supplementary motor area **(SMA)** (SMD = −1.26, 95% CI −1.99 to −0.54, *p* = 0.001) and primary motor cortex **(M1)** (SMD = −0.95, 95% CI −1.58 to −0.32, *p* = 0.003), while non-motor cortical targets did not reach significance (SMD = −0.43, 95% CI −1.04 to 0.19, *p* = 0.175). No significant between-subgroup differences were observed (Q_between_ = 3.19, *p* = 0.203). The subgroup analysis is presented in Fig. S19.

By stimulation frequency, significant effects were observed for 1 Hz (SMD = −1.73, 95% CI −3.00 to −0.45, *p* = 0.008) and 10 Hz (SMD = −0.82, 95% CI −1.29 to −0.36, *p* < 0.001), while the 50 Hz category (conventional 50 Hz rTMS and iTBS combined) did not reach significance (SMD = −1.00, 95% CI −2.52 to 0.52, *p* = 0.199). No significant between-subgroup differences were observed (Q_between_ = 1.71, *p* = 0.426). The subgroup analysis is presented in Fig. S20.

### 3.7 Sensitivity analysis

Leave-one-out analyses demonstrated that pooled estimates for the primary outcome (FOG-Q) and key secondary outcomes remained stable, confirming the robustness of the findings. An additional sensitivity analysis excluding cross-over trials yielded similar results. For outcomes with few contributing studies, significance was more sensitive to individual study removal, though the direction of effect remained consistent. Results are presented in Supplementary Figs. S2–S10.

### 3.8 Publication Bias

Publication bias was assessed for outcomes with at least ten contributing comparisons. For the primary outcome (FOG-Q change score), visual inspection of the funnel plot suggested asymmetry, and Egger's regression test was significant (intercept = −6.22, 95% CI −10.04 to −2.41, *t* = 3.63, one-tailed *p* = 0.002), indicating possible small-study effects. For UPDRS Part III motor scores, Egger's test did not reach statistical significance (intercept = −4.97, 95% CI −11.70 to 1.75, *t* = 1.67, one-tailed *p* = 0.064), although some visual asymmetry could not be excluded. The corresponding funnel plots are presented in Supplementary Figs. S11–S12.

### 3.9 Trial sequential analysis

Trial sequential analysis for the primary outcome demonstrated that the cumulative *Z*-curve crossed the trial sequential monitoring boundary despite the relatively modest cumulative sample size, suggesting that the observed benefit of rTMS on FOG-Q scores is unlikely to represent a spurious false-positive finding due to random error. The heterogeneity-adjusted required information size was estimated at 192 participants. The TSA plot is presented in Supplementary Fig. S21.

### 3.10 Certainty of evidence

Using the GRADE framework, the certainty of evidence for the primary outcome (FOG-Q change score) was rated as moderate. The evidence was downgraded one level for imprecision due to the relatively small total sample size and wide confidence intervals. No further downgrading was applied for risk of bias, inconsistency, indirectness, or publication bias.

### 3.11 Safety outcomes

No deaths or increases in fall incidence associated with rTMS were reported. Adverse events were infrequent and mild, including transient headache and scalp discomfort. Due to inconsistent reporting across trials, quantitative synthesis was not performed. A summary is presented in Supplementary Table S1.

## 4 Discussion

Our updated systematic review and meta-analysis of 12 RCTs involving 293 patients found that repetitive transcranial magnetic stimulation (rTMS) significantly improves freezing of gait (FOG) severity in patients with Parkinson's disease (PD), as demonstrated by a robust reduction in FOG-Q scores (SMD = −0.97; *p* < 0.001). Our findings also indicate that rTMS provides secondary benefits in general motor function (UPDRS-III), functional mobility (TUG), and gait velocity. Remarkably, the intervention significantly improved turning parameters, including turn time and the number of steps required to turn. These results suggest that rTMS effectively modulates the complex neural networks responsible for gait initiation and transitions, which are typically resistant to standard dopaminergic therapy [32]. To place the primary statistical result in a clinically interpretable frame, the pooled standardized mean difference of −0.97 represents a large effect by conventional benchmarks (small 0.2, medium 0.5, large 0.8) [38]. The minimal clinically relevant change for freezing-of-gait severity has been estimated at approximately 3 points on the FOG score [39]; given typical FOG-Q standard deviations of roughly 3 to 5 points across the included trials, an effect of this magnitude corresponds to an improvement on the order of 3 to 5 points, plausibly meeting or exceeding the threshold for a perceptible, clinically meaningful change in freezing-related gait disability. This interpretation should nonetheless be viewed cautiously, given the perceived nature of the FOG-Q and the heterogeneity in baseline severity across trials.

Our study provides a more focused analysis compared to previous reviews [33], [34], [35] by prioritizing freezing of gait **(FOG)** as the primary outcome. In contrast to Liu et al. (2024) [33], which noted the highest effect for 25 Hz stimulation, our analysis of more recent data demonstrates that 1 Hz (SMD = −1.73) and 10 Hz (SMD = −0.82) protocols remain highly effective. Regarding stimulation site, our findings demonstrate the efficacy of targeting the Supplementary Motor Area (SMA) (SMD = −1.26) and the Primary Motor Cortex (M1) (SMD = −0.95). While other reviews [33], [36] reported that M1 stimulation yielded the largest effect size in their samples, the present data suggest that the SMA is also an effective target for alleviating freezing of gait **(FOG)**. This supports the hypothesis that the SMA plays a critical role in the temporal organization of movement and is a highly appropriate target for cortical stimulation in FOG-specific interventions [36].

Leave-one-out sensitivity analyses indicated that the pooled estimates for the FOG-Q and motor scores were not driven by any single trial, supporting the stability of the primary effect.

Despite these strengths, certain limitations must be acknowledged. Safety data across trials were often insufficient or inconsistent, precluding formal quantitative synthesis of adverse events. Additionally, although sensitivity analyses excluding cross-over trials yielded consistent results, the inclusion of diverse study designs introduces methodological variability that should be considered when interpreting the magnitude of the pooled effects. A related limitation is that the pooled and subgroup estimates combine trials that differed in stimulation target, frequency, number of sessions, and pulses per session. Because the number of trials within any single target-by-frequency combination was small, subgroup analyses by site and by frequency necessarily pooled similar frequencies across different targets, and similar targets across different frequencies. The resulting estimates should therefore be read as broad, hypothesis-generating signals rather than protocol-specific recommendations, and the per-trial protocol parameters, including number of sessions and pulses per session, are now reported in full (Table 1 and Supplementary Table S2) to allow readers to weigh this heterogeneity directly. Another limitation relates to the primary outcome measure. The FOG-Q is a subjective, patient-perceived instrument rather than an objective measure of freezing. It quantifies self-reported freezing-related gait disability through recall and rating of symptom frequency and severity, not directly observed or instrumented freezing behaviour. Such perceived measures are susceptible to recall bias, day-to-day symptom fluctuation, and the absence of blinding at the level of patient self-report, all of which may inflate or attenuate the apparent treatment effect. The instrument is also not exclusively restricted to freezing episodes; its first two items capture broader gait impairment and walking-related functional difficulty. Therefore, the pooled FOG-Q effect should be interpreted as reflecting improvement in patient-perceived, freezing-related gait disability rather than objectively verified reduction in freezing episodes. Future trials should complement FOG-Q or NFOG-Q scores with objective, instrumented assessments of freezing during standardized gait and turning tasks, such as gait-laboratory or wearable-sensor quantification of freezing frequency and duration. The total sample size was modest (*n* = 293), and individual trials were small, limiting statistical power. Long-term follow-up data were limited, with most studies reporting outcomes only up to 4 weeks post-intervention. The restriction to English-language publications may have introduced language bias.

Additionally, reporting of medication state during outcome assessment was inconsistent across the included trials, with several studies evaluating patients in the ON state, others in the OFF state, and a subset not explicitly specifying the assessment condition. Because freezing of gait severity is known to vary substantially between medication states, this represents an additional source of clinical heterogeneity. The available data did not support a reliable ON versus OFF subgroup synthesis, and future randomized controlled trials should standardize and explicitly report the medication-state context of all freezing assessments.

The certainty of evidence for the primary outcome was rated as moderate using the GRADE framework, primarily due to imprecision arising from the relatively small cumulative sample size, wide confidence intervals across several secondary outcomes, and variability in stimulation protocols and study designs. Future large-scale, adequately powered, multi-center randomized controlled trials with standardized stimulation parameters, clearly defined medication-state assessments, longer follow-up durations, and rigorous adverse-event reporting would be required to increase the certainty of evidence toward a high-confidence level.

From a clinical perspective, rTMS should be considered a viable and safe adjunctive therapy for Parkinson's disease **(PD)** patients experiencing freezing of gait **(FOG)**. Our findings suggest that clinicians should prioritize the SMA or M1 as primary stimulation targets, as these regions demonstrated significant improvements in FOG-Q scores, whereas non-motor targets did not reach statistical significance. Protocol selection can be tailored to patient tolerance and available institutional equipment [37], given that both high-frequency (10 Hz) and low-frequency (1 Hz) stimulation yielded robust and significant benefits. Given that freezing of gait **(FOG)** often remains refractory to standard dopaminergic treatment, the integration of rTMS into multimodal rehabilitation strategies could offer a critical pathway to enhancing functional mobility and quality of life. To address current heterogeneity, future research must prioritize large-scale, multi-center RCTs with standardized protocols [20], [37] and rigorous, explicit reporting of adverse events. Additionally, investigating the long-term durability of rTMS effects through extended follow-up periods is essential to determine its role in chronic disease management.

## 5 Conclusion

Repetitive transcranial magnetic stimulation is a safe and effective non-invasive intervention for patients with Parkinson's disease, significantly improving freezing of gait, motor function, functional mobility, and key gait parameters including turn time and turn steps. The effects are most pronounced when targeting motor-related cortical areas, specifically the supplementary motor area **(SMA)** and primary motor cortex **(M1)**, and are observed even in patients with advanced disease (mean disease duration 4–12 years). Both low-frequency (1 Hz) and high-frequency (10 Hz) protocols are effective, providing clinical flexibility. These findings, supported by moderate-certainty evidence, endorse the integration of rTMS into multimodal rehabilitation strategies for Parkinson's disease **(PD)** patients with freezing of gait **(FOG)**. Future large-scale trials with standardized protocols, longer follow-up, and rigorous safety reporting are warranted to further confirm the long-term efficacy and optimize clinical application of rTMS.

## CRediT authorship contribution statement

**Lara Hamzeh Hamzeh:** Writing – original draft, Methodology, Investigation, Conceptualization. **Yahya Kayed AbuJwaid:** Writing – original draft, Methodology, Formal analysis, Data curation. **Sara M.F. Fahmy:** Writing – original draft, Data curation. **Dania AbuHawas:** Writing – original draft, Investigation. **Habiba Tariq Saeed:** Writing – original draft, Investigation. **Daher Heib:** Writing – original draft, Data curation. **Mawatheeq Al-Yafrosi:** Writing – original draft, Data curation. **Ahmed Noureldeen Abbas:** Writing – original draft, Formal analysis, Data curation. **Karima El Refaei:** Writing – original draft, Formal analysis, Data curation. **Christian Cortes Armijo:** Writing – original draft. **Majd A. AbuAlrob:** Writing – review & editing, Visualization, Validation, Supervision, Methodology, Investigation, Conceptualization.

## Funding

This research received no external funding.

## Declaration of competing interest

The authors declare that they have no known competing financial interests or personal relationships that could have appeared to influence the work reported in this paper.
