# Supplementary material for: Repetitive transcranial magnetic stimulation for freezing of gait in Parkinson's disease: a systematic review and meta-analysis
Source: Clin Park Relat Disord. 2026 Jun 15;15:100465. doi: 10.1016/j.prdoa.2026.100465 (PMC13292664; doi:10.1016/j.prdoa.2026.100465)
Supplement: Supplementary file 1 — Supplementary material [file mmc1.docx]

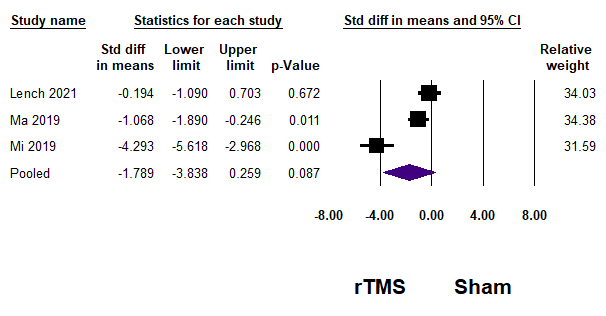


**Figure S1. Forest plot of the effect of repetitive transcranial magnetic stimulation (rTMS) on gait cadence compared with sham stimulation.** Effect sizes are expressed as standardized mean differences (SMDs) with 95% confidence intervals (CIs). Negative values favor rTMS.


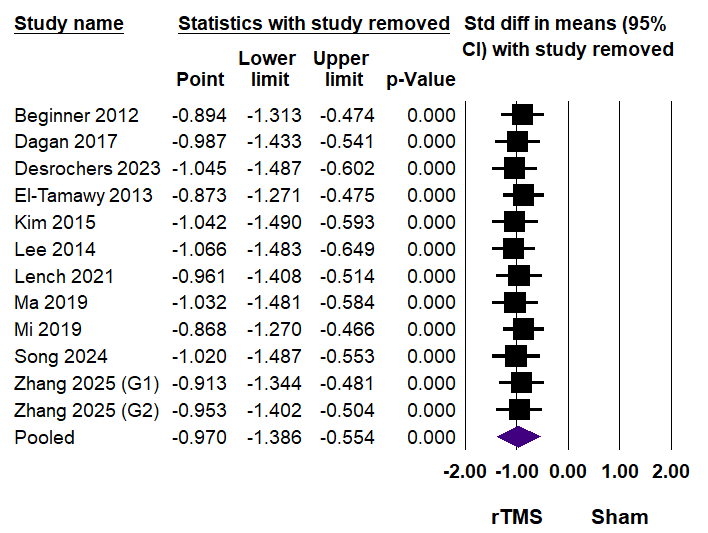


**Figure S2. Leave-one-out sensitivity analysis for the effect of rTMS on change in FOG-Q scores.**


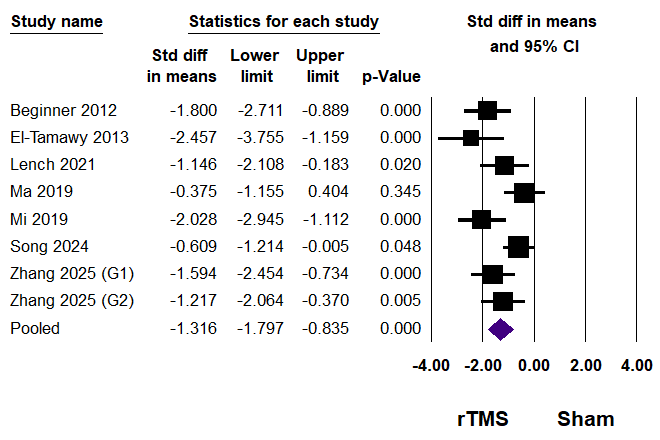


**Figure S3. Sensitivity analysis excluding cross-over trials for the effect of rTMS on change in FOG-Q scores.**


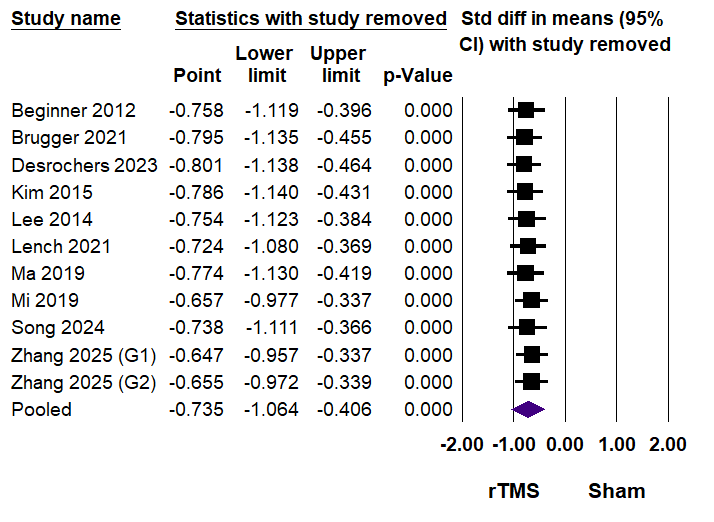


**Figure S4. Leave-one-out sensitivity analysis for the effect of rTMS on change in UPDRS Part III scores.**


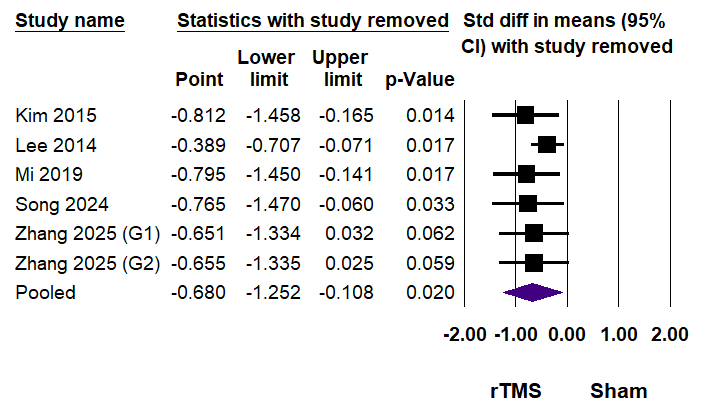


**Figure S5. Leave-one-out sensitivity analysis for the effect of rTMS on Timed Up and Go (TUG) performance.**


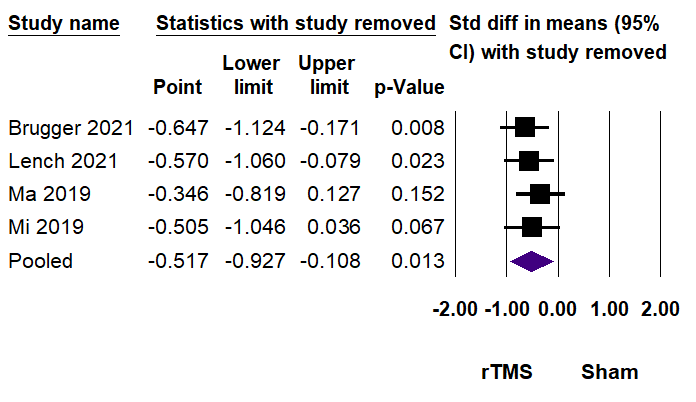


**Figure S6. Leave-one-out sensitivity analysis for the effect of rTMS on gait speed or velocity.**


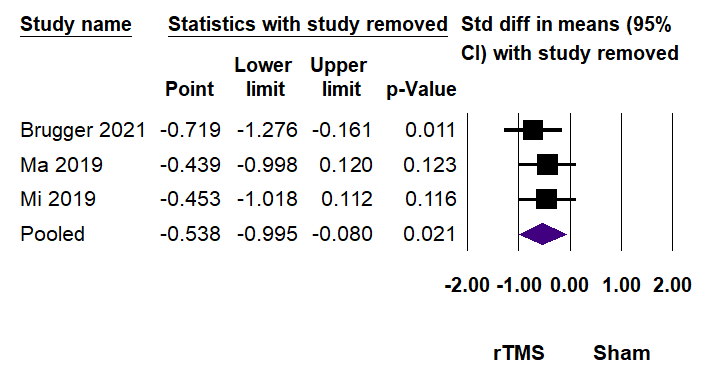


**Figure S7. Leave-one-out sensitivity analysis for the effect of rTMS on step or stride length.**


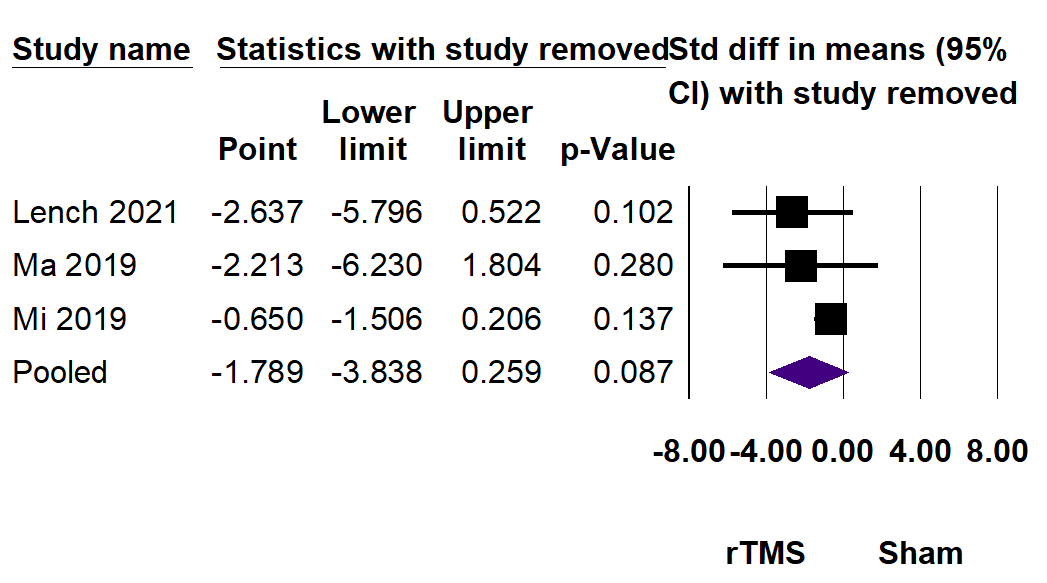


**Figure S8. Leave-one-out sensitivity analysis for the effect of rTMS on gait cadence (steps per minute).**


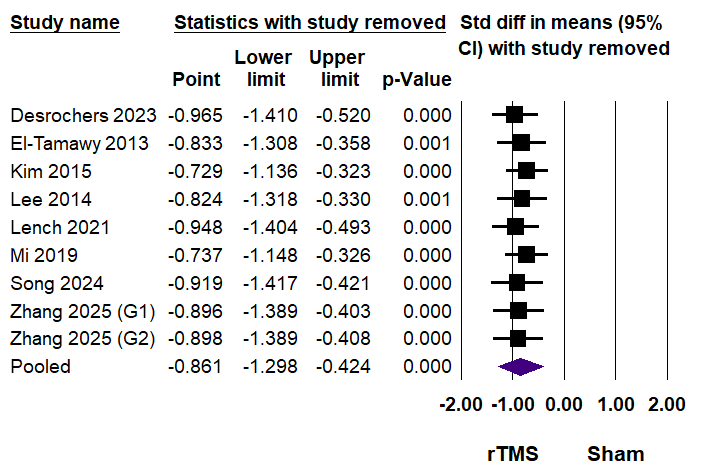


**Figure S9. Leave-one-out sensitivity analysis for the effect of rTMS on turn time (seconds).**


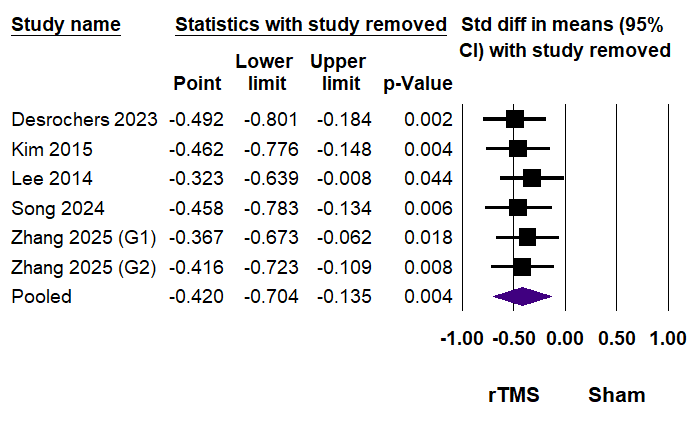


**Figure S10. Leave-one-out sensitivity analysis for the effect of rTMS on the number of steps required during turning.**


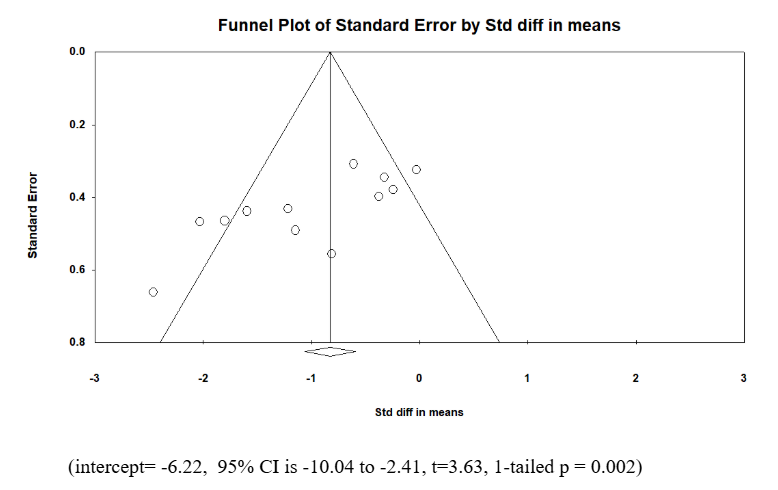


**Figure S11. Funnel plot for assessment of publication bias in the primary outcome (FOG-Q change scores). Egger’s regression test was significant (intercept = −6.22, 95% CI −10.04 to −2.41, t = 3.63, one-tailed p = 0.002), suggesting possible small-study effects.**


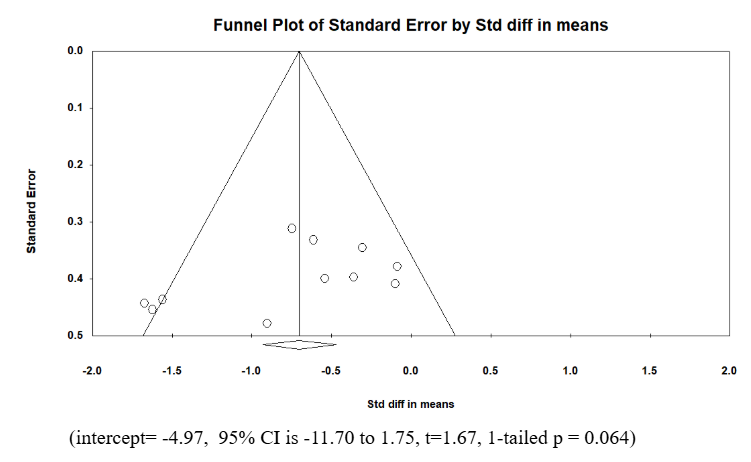


**Figure S12. Funnel plot for assessment of publication bias in motor outcomes measured by UPDRS-III scores. Egger’s regression test did not reach statistical significance (intercept = −4.97, 95% CI −11.70 to 1.75, t = 1.67, one-tailed p = 0.064).**

*
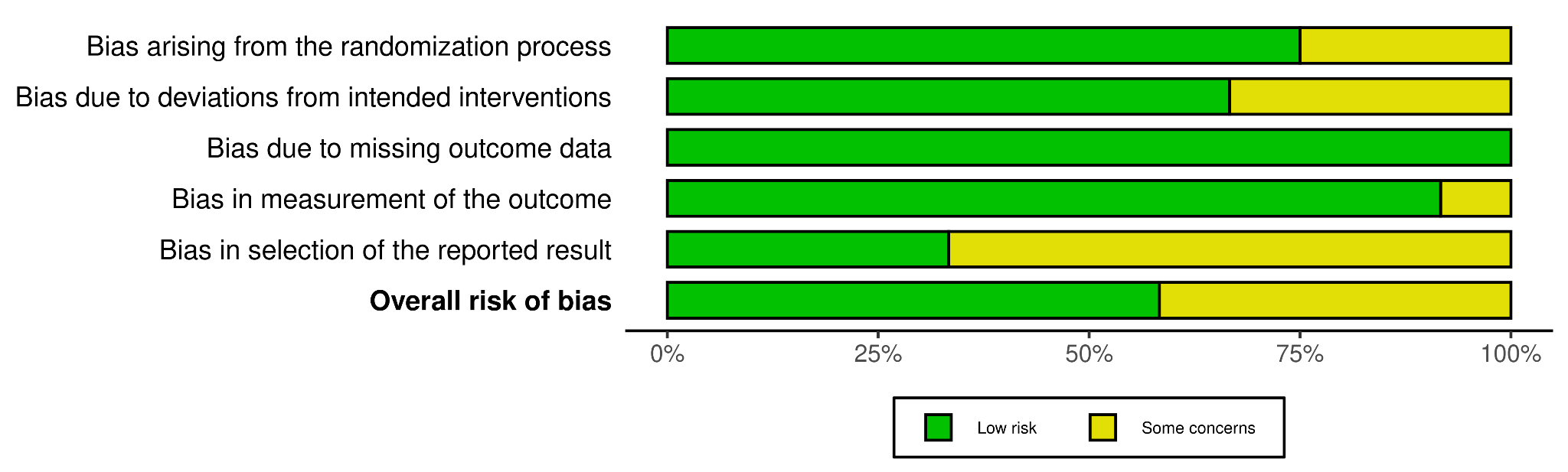
*

**Figure S13.** Summary of risk of bias judgments across all included studies, presented as the proportion of studies rated as low risk (green) or some concerns (yellow) for each domain of the Cochrane Risk of Bias 2 (RoB 2) tool. The domain with the highest proportion of low risk judgments was missing outcome data (100%), followed by measurement of the outcome (91.7%), the randomization process (75.0%), and deviations from intended interventions (66.7%). The domain with the most concerns was selection of the reported result, where only 33.3% of studies were judged as low risk, primarily due to the absence of pre-registered analysis plans.


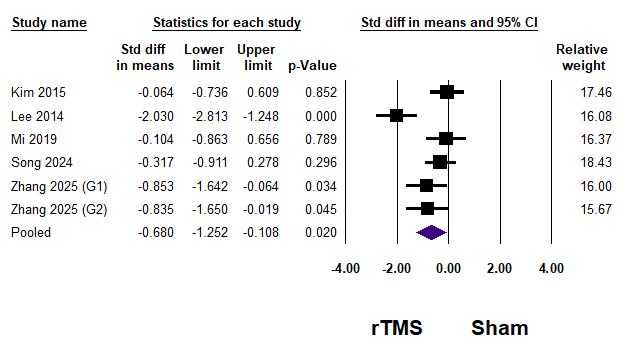


**Figure S14. Forest plot of the effect of repetitive transcranial magnetic stimulation (rTMS) on Timed Up and Go (TUG) performance compared with sham stimulation.** Effect sizes are expressed as standardized mean differences (SMDs) with 95% confidence intervals (CIs). Negative values favor rTMS. Zhang 2025 (G1) and Zhang 2025 (G2) represent separate intervention arms from the same study compared with the shared sham control group.


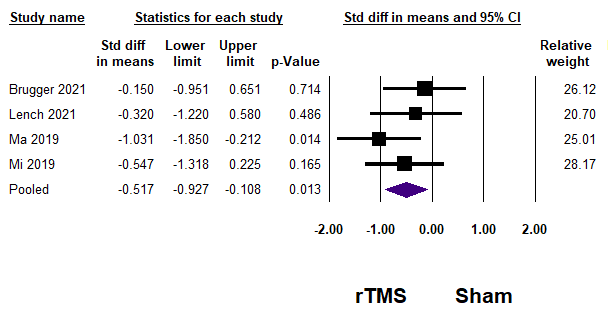


**Figure S15. Forest plot of the effect of repetitive transcranial magnetic stimulation (rTMS) on gait speed compared with sham stimulation.** Effect sizes are expressed as standardized mean differences (SMDs) with 95% confidence intervals (CIs). Negative values favor rTMS.


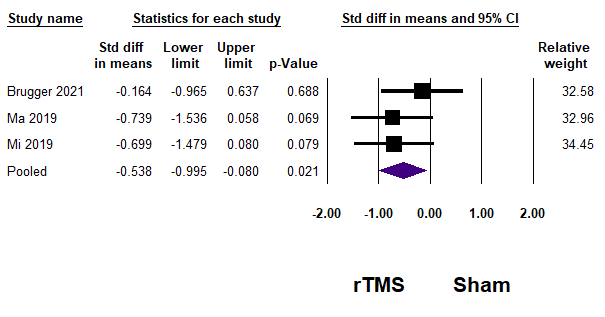


**Figure S16. Forest plot of the effect of repetitive transcranial magnetic stimulation (rTMS) on step or stride length compared with sham stimulation.** Effect sizes are expressed as standardized mean differences (SMDs) with 95% confidence intervals (CIs). Negative values favor rTMS.


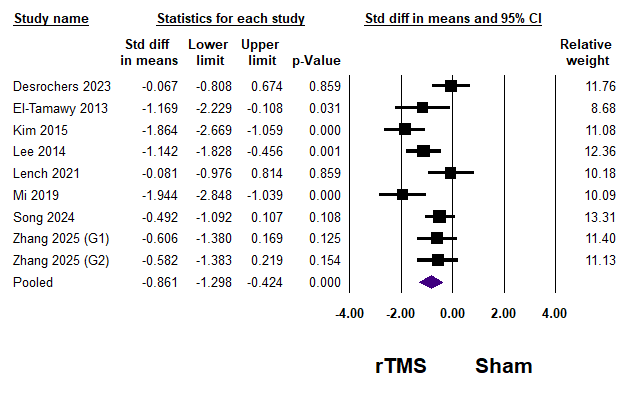


**Figure S17. Forest plot of the effect of repetitive transcranial magnetic stimulation (rTMS) on turn time compared with sham stimulation.** Effect sizes are expressed as standardized mean differences (SMDs) with 95% confidence intervals (CIs). Negative values favor rTMS. Zhang 2025 (G1) and Zhang 2025 (G2) represent separate intervention arms from the same study compared with the shared sham control group.


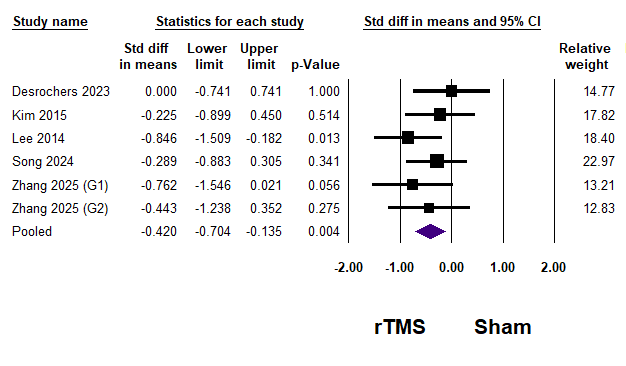


**Figure S18.** Forest plot of the effect of repetitive transcranial magnetic stimulation (rTMS) on the number of steps required during turning compared with sham stimulation. Effect sizes are expressed as standardized mean differences (SMDs) with 95% confidence intervals (CIs). Negative values favor rTMS. Zhang 2025 (G1) and Zhang 2025 (G2) represent separate intervention arms from the same study compared with the shared sham control group.


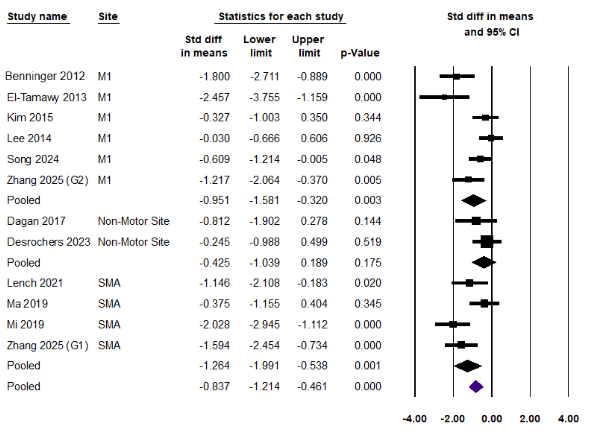


**Figure S19. Subgroup analysis of the primary outcome (FOG-Q change scores) according to stimulation site (M1, SMA, and non-motor cortical targets).**


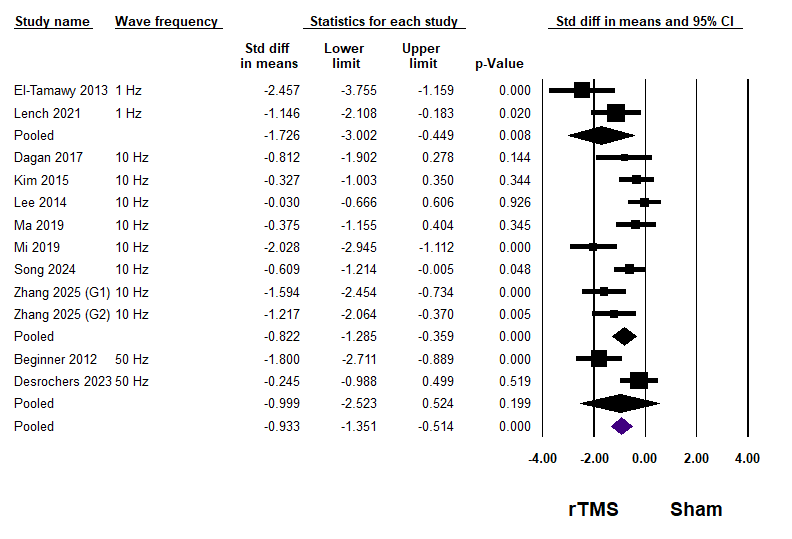


**Figure S20. Subgroup analysis of the primary outcome (FOG-Q change scores) according to stimulation frequency (1 Hz, 10 Hz, and 50 Hz).**


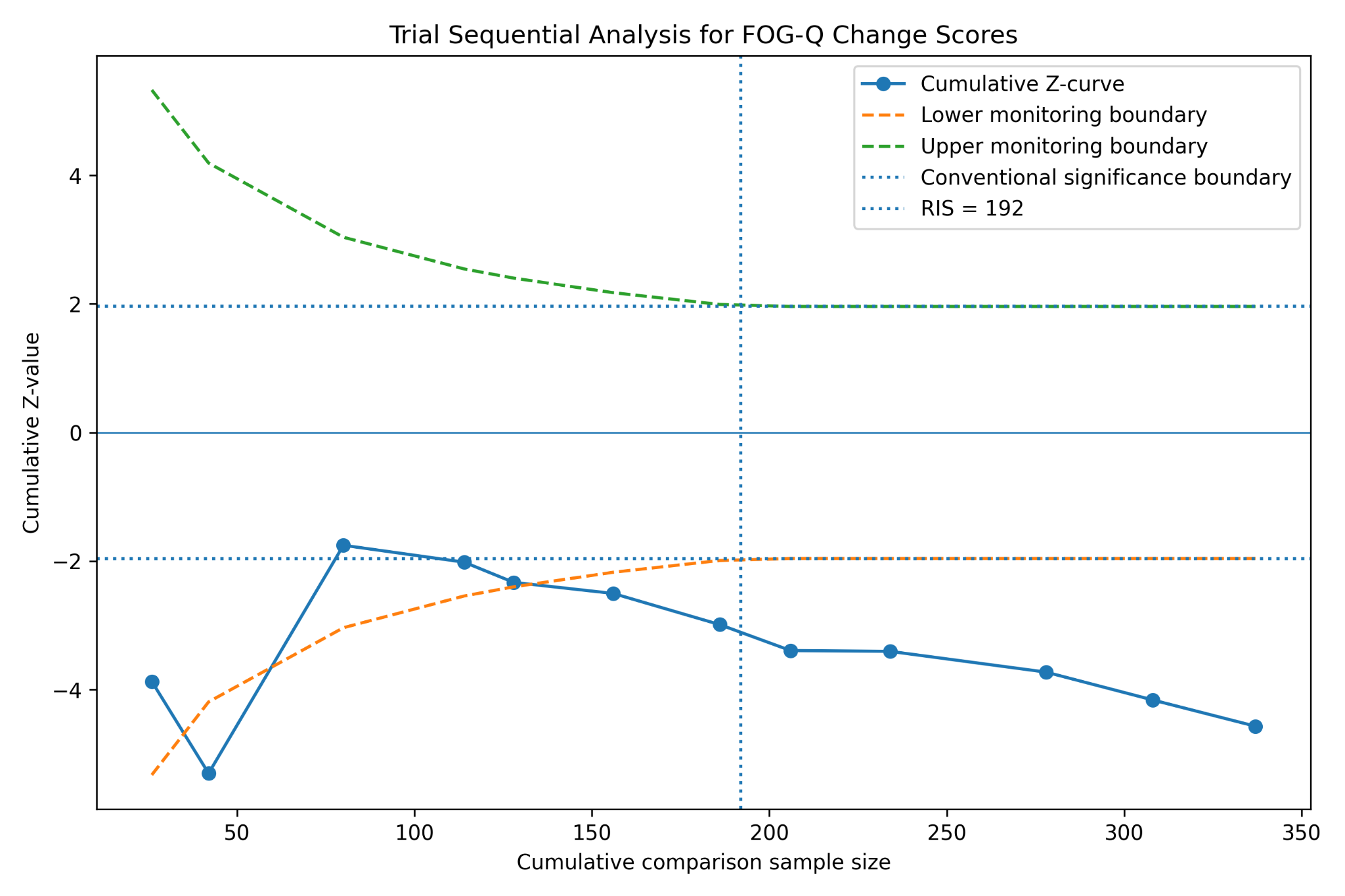


**Figure S21. Trial sequential analysis for the primary outcome (FOG-Q change score).**

**Table S1. Reported adverse events in studies included in the review.**

| Zhang 2025 | 5 |
| --- | --- |
| El-Tamawy 2013 | 3 |
| Kim 2015 | 1 |
| Lee 2014 | 1 |
| Total | 10 |

**Supplementary Table S2.** *Stimulation protocol parameters and trial-level freezing-of-gait outcomes for each included study.*

| **Study ID** | **Stimulation target** | **Frequency / mode** | **Sessions** | **Pulses per session** | **Total pulses** | **FOG-Q outcome** |
| --- | --- | --- | --- | --- | --- | --- |
| Benninger 2012 | M1 | 50 Hz rTMS | 8 (over 2 wk) | NR | NR | Improved; favoured rTMS |
| Brugger 2021 | SMA | iTBS (50 Hz triplets / 5 Hz) | NR | NR | NR | Improved; favoured iTBS |
| Dagan 2017 | PFC | 10 Hz deep rTMS | NR | NR | NR | Improved; favoured rTMS |
| Desrochers 2023 | PPC | iTBS (50 Hz triplets / 5 Hz) | NR | NR | NR | No significant change |
| El-Tamawy 2013 | M1 | 1 Hz rTMS | NR | NR | NR | Improved; favoured rTMS |
| Kim 2015 | M1 (M1-LL) | 10 Hz rTMS | 5 (1 wk) | 1000 | 5000 | Improved; favoured rTMS |
| Lee 2014 | M1 | 10 Hz rTMS | NR | NR | NR | Improved; favoured rTMS |
| Lench 2021 | SMA | 1 Hz rTMS | 10 | 1200 | 12000 | Improved; favoured rTMS |
| Ma 2019 | SMA | 10 Hz rTMS | NR | NR | NR | Improved; favoured rTMS |
| Mi 2019 | SMA | 10 Hz rTMS | 10 (2 wk) | 1000 | 10000 | Improved; favoured rTMS |
| Song 2024 | M1 (bilateral) | 10 Hz rTMS | 10 | NR | NR | Improved; favoured rTMS |
| Zhang 2025 | M1 and SMA | 10 Hz rTMS | 10 (daily) | NR | NR | Improved; favoured rTMS |

FOG-Q, Freezing of Gait Questionnaire; iTBS, intermittent theta-burst stimulation (50 Hz triplets repeated at 5 Hz); M1, primary motor cortex; M1-LL, lower-limb representation of the primary motor cortex; NR, not reported in the source publication; PFC, prefrontal cortex; PPC, posterior parietal cortex; rTMS, repetitive transcranial magnetic stimulation; SMA, supplementary motor area. FOG-Q outcome denotes the direction of the between-group effect at the primary post-intervention timepoint.
